# Predictors for unfavorable prognosis after stroke with perforator artery disease

**DOI:** 10.3389/fneur.2024.1340085

**Published:** 2024-01-24

**Authors:** Linghua Song, Xiaoli Lou, Wenhao Han, Lihui Yang, Siping Guo, Yasi Jiang, Hao Peng, Yonggang Hao

**Affiliations:** ^1^Suzhou Dushu Lake Hospital, Suzhou, Jiangsu, China; ^2^School of Public Health, Soochow University Medical College, Suzhou, Jiangsu, China

**Keywords:** perforator artery disease, neutrophil-to-lymphocyte ratio, unfavorable prognosis, Chinese ischemic stroke subclassification (CISS), modified Rankin Scale

## Abstract

**Background and purpose:**

Perforator artery disease (PAD) is an important subtype of ischemic stroke. The risk factors affecting the prognosis of patients with PAD are unclear. This study aimed to investigate the risk factors affecting the unfavorable prognosis of PAD.

**Methods:**

Patients with PAD were enrolled from Dushu Lake Hospital Affiliated to Soochow University and diagnosed as stroke with PAD during the period from September 2021 to July 2023 and followed up with a modified Rankin Scale (mRS) after 90 days, defining the mRS of 0–2 as a group with favorable prognosis, and 3–6 as a group with unfavorable functional outcome. Logistic regression was used to identify predictors for PAD. Multiple logistic regression analysis and receiver operating characteristics (ROC) were used to identify predictors of unfavorable prognosis.

**Results:**

Of the 181 enrolled patients, 48 (26.5%) were identified with unfavorable prognosis. On multivariate analysis, increased age (*OR* = 1.076, 95% *CI*: 1.012 ~ 1.144, *p* = 0.019), higher National Institutes of Health Stroke Scale (NIHSS) score at admission (*OR* = 2.930, 95% *CI*: 1. 905 ~ 4.508, *p* < 0.001), and increased neutrophil-to-lymphocyte ratio (NLR) (*OR* = 3.028, 95% *CI*: 1.615 ~ 5.675, *p* = 0.001) were independent risk factors for unfavorable prognosis in patients with PAD, and the area under the receiver operating characteristic curve was 0.590, 0.905, and 0.798, and the multi-factor diagnostic model (Model 2) showed reliable diagnostic specificity and sensitivity (area under the curve = 0.956, *p* < 0.001, specificity 0.805, sensitivity 0.958, accuracy 0.845).

**Conclusion:**

Increased baseline NLR and NIHSS score and aging may be independent risk factors for unfavorable prognosis of patients with PAD. NLR can be used as a potential biological indicator to predict the prognosis of stroke with PAD.

## Introduction

Perforator artery disease (PAD), which accounts for approximately one-quarter of all ischemic strokes, was defined as an acute isolated infarct in the clinically relevant territory of perforator arteries such as the basal ganglia region and brainstem (1). According to the CISS typology, the probable pathogenesis of PAD includes atherosclerosis at the proximal segment of the penetrating arteries and lipohyalinotic degeneration of arterioles (2, 3). The diagnostic criteria include the following: (1) acute isolated infarct in the clinically relevant territory of one penetrating artery, regardless of the size of the infarct; (2) no evidence of atherosclerotic plaque (detected by HR-MRI) or any degree of stenosis in the parent artery (detected by TCD, MRA, CTA, or DSA); (3) with evidence of vulnerable plaques or stenosis ≥50% in ipsilateral proximal intracranial or extracranial large arteries, or cardiac disease that has a potential for embolism is classified in UE (multiple etiology); (4) other possible causes have been excluded. Due to different anatomical structures, patients with PAD also differ from those with large artery atherosclerosis in terms of pathogenesis, clinical features, and prognosis (4). Previous studies have shown that patients with PAD are prone to early neurological deterioration (END) and progressive movement disorders (PMD) (5, 6), but the risk factors affecting the prognosis of patients with PAD are unclear. Studies have suggested that neutrophil-to-lymphocyte ratio (NLR) affects post-thrombolysis early neurological outcomes in patients with large-vessel occlusive stroke (7, 8), but the effect on patients with PAD is rarely reported. Using the data from Dushu Lake Hospital affiliated to Soochow University, we analyzed the potential predictors for unfavorable outcomes in patients with PAD, as well as the correlation between NLR, an indicator of inflammation, and the prognosis of PAD and its clinical application value.

## Materials and methods

### Study participants

**Figure 1:**
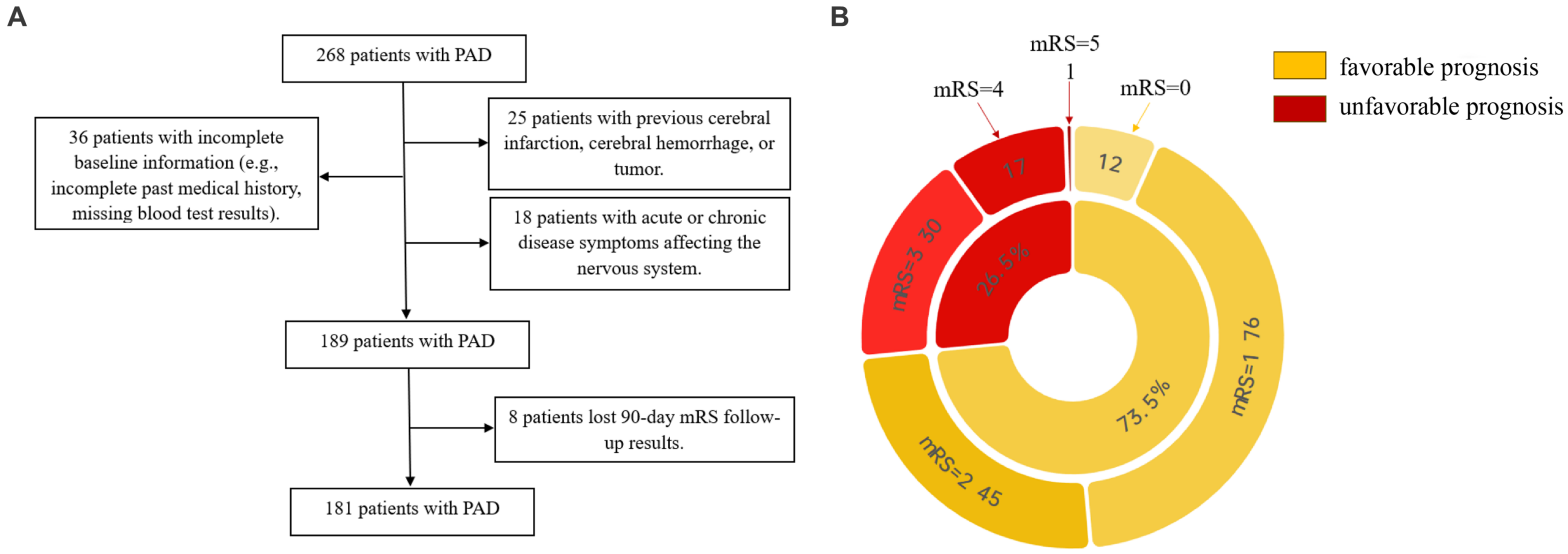
**(A)** Shows the process of the selection of participants. **(B)** Shows the distribution of prognostic subgroups according to mRS.

We retrospectively analyzed acute ischemic stroke patients with PAD who were hospitalized in Dushu Lake Hospital affiliated to Soochow University during the period from September 2021 to July 2023. Inclusion criteria: (1) meet the diagnostic criteria of the 2018 Guidelines for the Early Management of Patients With Acute Ischemic Stroke (9), magnetic resonance imaging (MRI) clearly defined intracranial new-onset single infarct lesion, which was compatible with the patient’s clinical symptoms and signs. As for etiological cause, perforator artery occlusion was considered according to the CISS classification; (2) first onset of the disease, within 72 h from onset to admission; and (3) age ≥ 18 years. Exclusion criteria: (1) patients with previous cerebral infarction, cerebral hemorrhage, or brain tumor; (2) patients with combined acute and chronic infectious diseases, autoimmune diseases, hematological disorders, severe cardiac, hepatic, renal diseases, and malignant tumors; (3) patients taking immunosuppressant, hormone, antibiotic, and antiplatelet aggregation drugs in the last 6 months; and (4) patients with surgical procedures in the last 6 months. The flowchart in Figure 1A shows the process of the selection of participants.

### Data collection

All the participants underwent standard assessments of demographic characteristics (age, sex, and body mass index [BMI]), vascular risk factors (hypertension, diabetes mellitus, dyslipidemia, current smoking, and current drinking), clinical assessment (blood pressure, treatment with IV thrombolysis, and NIHSS score), lesion location, and laboratory data. Systolic blood pressure (SBP) and diastolic blood pressure (DBP) were measured and recorded immediately after admission. Computed tomography, magnetic resonance, electrocardiogram, echocardiography, carotid ultrasonography, and transcranial Doppler were performed to assess the lesion location and confirm the stroke subtype. Laboratory data included total cholesterol (TC), triglyceride (TG), high-density lipoprotein (HDL), low-density lipoprotein (LDL), homocysteine (HCY), total leukocyte counts (WBC), neutrophil counts (NE), lymphocyte counts (LYM), and neutrophil-to-lymphocyte ratio (NLR). We conducted a follow-up observation using a 90-day modified Rankin Scale (mRS) (10) and further made the definition of the mRS of 0–2 as the favorable prognosis group, and 3–6 as the unfavorable prognosis group. The pie diagram in Figure 1B shows the distribution of prognostic subgroups according to mRS.

According to the intracranial arteries originating from the blood-supplying arteries at the site of the lesion, they were divided into anterior and posterior circulation groups: anterior circulation: an intracranial segment of the internal carotid artery, middle cerebral artery, anterior cerebral artery, ophthalmic artery, and anterior choroidal artery; and posterior circulation: posterior cerebral artery, basilar artery, and intracranial segment of the vertebral artery (11).

### Statistical analysis

Categorical variables were expressed as n (%), and continuous variables were expressed as means (standard deviation, SD) or medians (interquartile range, IQR). Continuous numerical variables were tested for normality using the Shapiro–Wilk test; if they did not conform to normal distribution, they were expressed as medians (quartiles). Comparison of quantitative data of multiple groups was analyzed using the one-way ANOVA, and the Kruskal–Wallis test was used for those who did not meet the conditions of the one-way ANOVA; comparison of normal qualitative data was tested using the chi-square test. Multifactorial analysis was analyzed using the logistic regression model, and *p* < 0.05 was considered statistically significant. Statistical analyses were performed using SPSS 26.0 (IBM, Armonk, NY) and Origin 2022.

## Results

**Figure 2:**
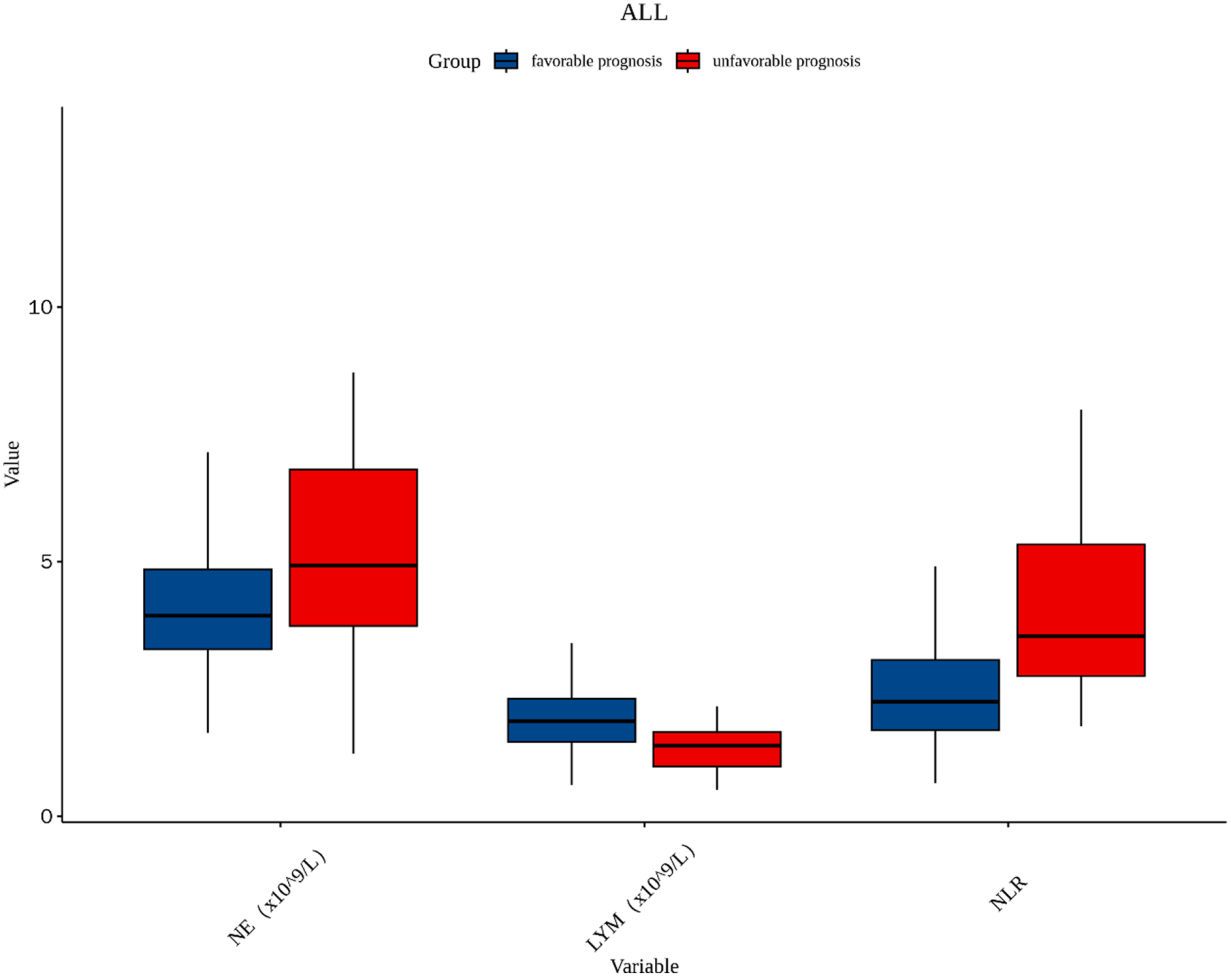
The boxplot in the distribution of inflammatory indices between two different prognosis outcome groups. NE, neutrophil; LYM, lymphocyte; NLR, neutrophil-to-lymphocyte ratio.

A total of 181 patients with PAD were chosen according to the inclusion and exclusion criteria, comprising 112 male patients and 69 female patients, of which 133 patients were in the favorable prognosis group and 48 patients were in the unfavorable prognosis group. The demographics and clinical characteristics of the two groups are shown in Table 1. Univariate logistic analysis showed that patients in the unfavorable prognosis group were older (median 66 versus 63, *p* = 0.019) and NIHSS scores at admission (median 5 versus 2, *p* < 0.001), baseline NE (median 4.93 versus 3.94, *p* = 0.003), and NLR were higher (median 3.53 versus 2.25, *p* < 0.001) than those in the favorable prognosis group, whereas the baseline LYM was lower (mean 1.35 versus 1.92, *p* < 0.001) in the unfavorable prognosis group. There were no statistical differences between the two groups in terms of other demographic characteristics, vascular risk factors, clinical assessment, lesion location, and laboratory data (*p* > 0.050). Figure 2 shows the boxplots of inflammatory indices between the two groups.

**Table 1:** Demographics and clinical characteristics of the subgroup according to prognosis.

Variable	All participants (*n* = 181)	Favorable prognosis (*n* = 133)	Unfavorable prognosis (*n* = 48)	*p-*value
Demographic characteristics				
Male, *n* (%)	112 (60.9%)	84 (62.2%)	28 (57.4%)	0.556
Age, years	64 (56 ~ 70)	63 (56 ~ 69)	66 (56 ~ 74)	0.019^*^
BMI (kg/m^2^)	24.00 (22.21 ~ 26.12)	24.52 ± 3.56	23.45 ± 2.22	0.055
Vascular risk factors, *n* (%)				
Hypertension	150 (82.8%)	111 (83.5%)	39 (81.3%)	0.728
Diabetes mellitus	61 (33.7%)	40 (30.0%)	21 (43.8%)	0.088
Dyslipidemia	50 (27.6%)	41 (30.8%)	9 (18.8%)	0.113
Current smoking	47 (27%)	37 (27.8%)	10 (20.8%)	0.346
Current drinking	30 (16.6%)	22 (16.5%)	8 (16.7%)	0.984
Clinical assessment				
NIHSS, score	2 (1 ~ 4)	2 (1 ~ 3)	5 (3 ~ 7)	<0.001^*^
SBP, mmHg	149.99 ± 20.88	148.31 ± 21.21	154.65 ± 19.38	0.074
DBP, mmHg	86.15 ± 11.98	84 (79 ~ 93)	88 (78 ~ 95)	0.806
Treatment with IV thrombolysis, *n* (%)	10 (5.5%)	6 (4.5%)	4 (8.3%)	0.328
Lesion location, *n* (%)				
Anterior circulation	104 (57.5%)	74 (55.6%)	30 (62.5%)	0.411
Posterior circulation	77 (42.5%)	59 (44.4%)	18 (37.5%)	
Laboratory data				
TC, mmol/L	4.31 (3.55 ~ 5.00)	4.35 (3.54 ~ 4.98)	4.12 (3.55 ~ 5.04)	0.525
TG, mmol/L	1.30 (0.96 ~ 1.96)	1.41 (1.01 ~ 2.02)	1.16 (0.83 ~ 1.49)	0.248
LDL, mmol/L	2.69 (1.97 ~ 3.34)	2.67 (2.02 ~ 3.36)	2.70 (1.95 ~ 3.28)	0.696
HDL, mmol/L	1.05 (0.88 ~ 1.31)	1.02 (0.87 ~ 1.27)	1.13 (0.93 ~ 1.34)	0.073
HCY, μmol/L	13.03 (10.60 ~ 18.23)	12.99 (10.66 ~ 17.57)	13.26 (10.32 ~ 19.66)	0.949
WBC, ×10^9/L	6.50 (5.45 ~ 7.99)	6.36 (5.43 ~ 7.55)	6.83 (5.44 ~ 8.62)	0.097
NE, ×10^9/L9/L	4.15 (3.35 ~ 5.29)	3.94 (3.28 ~ 4.86)	4.93 (3.70 ~ 6.86)	0.003^*^
LYM, ×10^9/L	1.71 (1.32 ~ 2.15)	1.92 ± 0.61	1.35 ± 0.49	<0.001^*^
NLR	2.57 (1.86 ~ 3.49)	2.25 (1.68 ~ 3.07)	3.53 (2.74 ~ 5.36)	<0.001^*^

BMI, body mass index; NIHSS, National Institute of Health Stroke Scale; SBP, systolic blood pressure; DBP, diastolic blood pressure; TC, total cholesterol; TG, cholesterol triglyceride; LDL, low-density lipoprotein; HDL, high-density lipoprotein; HCY, homocysteine; WBC, white blood cell; NE, neutrophil; LYM, lymphocyte; NLR, neutrophil-to-lymphocyte ratio.

Table 2 displays the results of the multiple logistic regression models for subgroups according to prognosis. Due to the correlation between NE, LYM, and NLR, we divided into two models for analysis. Furthermore, we incorporated potential risk factors of BMI, diabetes mellitus, HDL, SBP, and WBC (*p* < 0.1). In addition, due to the correlation between WBC NE and LYM, in Model 1, we removed the confounder WBC. After adjustment for all potential confounders, Model 1 showed that age (odds ratio [OR], 1.092; 95% confidence interval [CI] 1.019 ~ 1.171, *p* = 0.013), NIHSS (OR, 3.123; 95% CI 1.975 ~ 4.938, *p* < 0.001), NE (OR, 1.889; 95% CI 1.200 ~ 2.975, *p* = 0.006), and LYM (OR, 0.054; 95% CI 0.011 ~ 0.257, *p* < 0.001) were identified as independent factors for unfavorable prognosis for patients with PAD, while Model 2 also showed that age (OR, 1.076; 95% CI 1.012 ~ 1.144, *p* = 0.019), NIHSS (OR, 2.930; 95% CI 1.905 ~ 4.508, *p* < 0.001), and NLR (OR, 3.028; 95% CI 1.6154 ~ 5.675, *p* = 0.001) were identified as independent factors for unfavorable prognosis for patients with PAD.

**Table 2:** Multinomial logistic regression models for subgroups according to mRS.

Variable	OR	95% CI	*p-*value
Model 1			
Age	1.092	1.019 ~ 1.171	0.013*
BMI	0.946	0.773 ~ 1.159	0.592
Diabetes mellitus	0.362	0.108 ~ 1.216	0.100
SBP	1.025	0.966 ~ 1.056	0.093
HDL	1.413	0.184 ~ 10.849	0.740
NIHSS, score	3.123	1.975 ~ 4.938	<0.001*
NE	1.889	1.200 ~ 2.975	0.006*
LYM	0.054	0.011 ~ 0.257	<0.001*
Model 2			
Age	1.076	1.012 ~ 1.144	0.019*
BMI	0.942	0.779 ~ 1.139	0.535
Diabetes mellitus	0.337	0.106 ~ 1.072	0.066
SBP	1.027	0.998 ~ 1.056	0.073
HDL	1.759	0.650 ~ 13.646	0.589
WBC	0.884	0.831 ~ 1.203	0.434
NIHSS, score	2.930	1.905 ~ 4.508	<0.001*
NLR	3.028	1.615 ~ 5.675	0.001*

BMI, body mass index; SBP, systolic blood pressure; HDL, high-density lipoprotein; NIHSS, National Institute of Health Stroke Scale. NE, neutrophil; LYM, lymphocyte; NLR, neutrophil-to-lymphocyte ratio.

**Figure 3:**
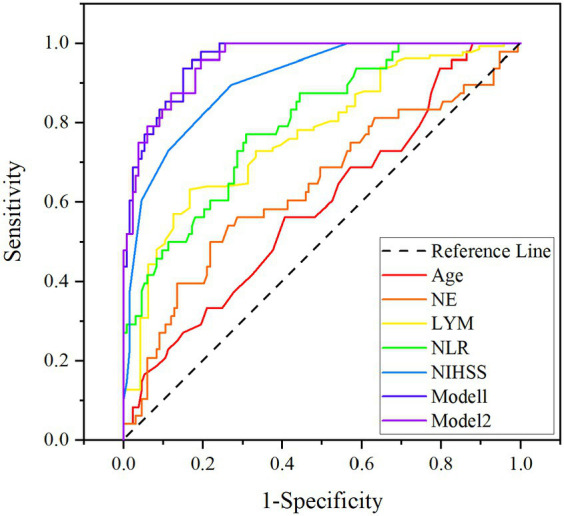
ROC curves for age, NIHSS on admission, NE, LYM, NLR, Model 1 (age, NIHSS, NE, and LYM), and Model 2 (age, NIHSS, and NLR). NIHSS, National Institute of Health Stroke Scale; NE, neutrophil; LYM, lymphocyte; NLR, neutrophil-to-lymphocyte ratio.

The ROC curves, which were depicted in Figure 3, were used to test the overall discriminative ability of risk factors for unfavorable prognosis for patients with PAD. We observed in Model 1 that the areas under the curve (AUC) of NE and LYM were 0.636 (95% CI, 0.538–0.734) and 0.768 (95% CI, 0.693–0.843) and the composite diagnostic model was 0.960 (95% CI, 0.935–0.984), and in Model 2, the area under the curve (AUC) of NLR was 0.798 (95% CI, 0.727–0.869) and the composite diagnostic model was 0.956 (95% CI, 0.930–0.982). In the performance of predicting prognosis of PAD, the AUC of NLR was superior to NE (0.798 versus 0.636, *p* < 0.001) and LYM (0.798 versus 0.768, *p* < 0.001), which means that NLR may be a better biological indicator to predict the prognosis of stroke with PAD. We also established optimal cutoff values at which the Youden Index was highest. The details are described in Supplementary Table S1.

Furthermore, patients with PAD were divided into the anterior circulation group (*n* = 104) and posterior circulation group (*n* = 77) according to the lesion site shown on the images, and the changes of inflammatory indices NE, LYM, and NLR were analyzed to see whether there was any difference between the two groups. The results shown in Table 3 suggested that the decrease in LYM and the increase in NLR in the anterior circulation group were significantly correlated with an unfavorable prognosis and that the increase in NE and NLR as well as the decrease in LYM in the posterior circulation group were all significantly correlated with an unfavorable prognosis (*p* > 0 05).

**Table 3:** Subgroup analysis of anterior and posterior circulation.

Variable	Favorable prognosis	Unfavorable prognosis	*P-*value		OR
Anterior circulation (*n* = 104)				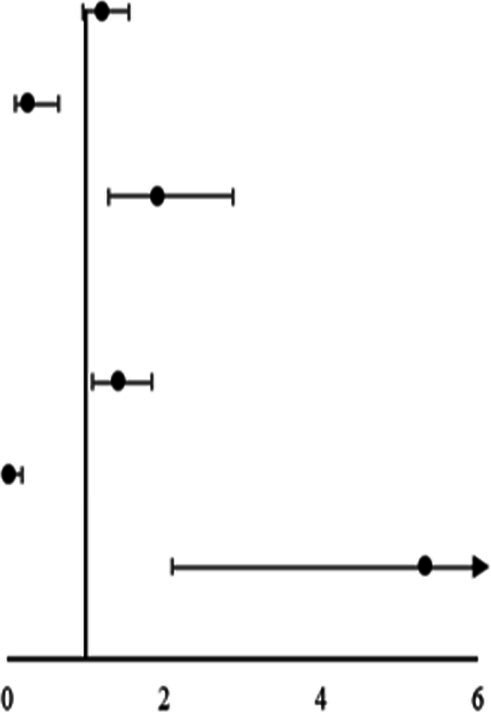	
NE	4.00 (3.28 ~ 5.28)	4.93 (3.55 ~ 5.95)	0.104		1.219 (0.960 ~ 1.547)
LYM	1.87 ± 0.58	1.50 ± 0.43	0.004^*^		0.264 (0.106 ~ 0.655)
NLR	2.39 (1.76 ~ 3.12)	3.10 (2.46 ~ 3.99)	0.001^*^		1.416 (1.088 ~ 1.842)
Posterior circulation(n = 77)					
NE	3.94 (3.19 ~ 4.60)	5.72 ± 2.61	0.010^*^		0.027 (0.004 ~ 0.193)
LYM	1.94 (1.44 ~ 2.45)	0.99 (0.84 ~ 1.38)	<0.001^*^		5.329 (2.113 ~ 13.441)
NLR	2.25 ± 0.87	4.91 (3.15 ~ 7.43)	<0.001^*^		1.928 (1.288 ~ 2.886)

NE, neutrophil; LYM, lymphocyte; NLR, neutrophil-to-lymphocyte ratio.

## Discussion

Our study showed that increasing age, higher NIHSS score at admission, decreased LYM, and elevated NE and NLR were significantly associated with unfavorable prognosis in patients with PAD. Further multifactorial analysis showed that elevated NLR was independently associated with an unfavorable prognosis. Further subgroup analysis showed that NLR correlated with unfavorable prognosis in both anterior and posterior groups, which suggests that inflammation and immune factors may play an essential role in the clinical regression process of PAD and the use of NLR to predict prognosis in PAD deserves further investigation.

Aging exacerbates the damage and dysfunction of different components of the non-injurious brain unit, affecting the integrity of the neurovascular unit and the vulnerability to neurodegeneration, thus accelerating the progression and deterioration of brain injury (12). The National Institute of Health Stroke Scale (NIHSS) is currently the most used clinical scale for assessing neurologic deficits. Studies have demonstrated that the more severe a patient’s neurological deficit, the higher the risk of poor prognosis (13). This study further confirms that aging and elevated baseline NIHSS are risk factors for unfavorable prognosis of PAD.

Acute ischemic stroke triggers an inflammatory response at an early stage, leading to the disruption of the blood–brain barrier and the deterioration of neurological function (14, 15). Studies have shown that neutrophils promote atherogenesis and the progression of atherosclerosis by mediating a non-specific inflammatory response (16, 17), and the infiltration of neutrophils can cause an increase in cerebral infarct volume and worsening of ischemic brain damage (18). Whereas lymphocytes have a role in maintaining immune homeostasis and immune tolerance, they can repair damage through inflammatory responses (19, 20). Tregs in lymphocytes are key cerebroprotective immunomodulators in acute stroke (21), which act as endogenous neuroprotective modulators in maintaining blood–brain barrier permeability, delaying leukocyte infiltration, reducing tissue edema, decreasing brain damage, and playing a protective role in improving prognostic recovery (22–24). Lack of lymphocytes can accelerate the process of atherosclerosis progression (25, 26). Previous studies have found that there is a significant tendency for lymphocytes to decrease at the onset of cerebral infarction (27, 28), and although immunosuppression may reduce brain damage, it may impede neuronal cell repair and increase the risk of systemic bacterial infections during the acute phase of the disease (29, 30). Elevated levels of NLR have been observed in diabetic patients who develop renal arterial vitreous degeneration (31, 32), while lipohyalinotic degeneration is the main pathogenesis of PAD, so we hypothesized that elevated NLR plays a role in the development of PAD and may be involved in the progression of penetrating arterial lesions. In the present study, we observed that elevated NLR was independently associated with unfavorable functional outcomes in PAD, suggesting that mediating the inflammatory response could be a new target for the prevention and treatment of PAD.

As a retrospective study, our study has some limitations. First, this study only collected data on baseline inflammatory markers after emergency or admission and did not assess the dynamic changes in NLR during hospitalization, which may provide information for discovering the pathophysiological mechanisms of the inflammatory response after the onset of PAD (33). Second, the single-center study was not fully representative of the total patients with PAD. Moreover, the sample size is small, and there is a possibility of bias in some clinical characteristics. Therefore, more prospective, large-sample, multicenter studies are needed for further validation. Despite these limitations mentioned above, it is the first time the relationship between inflammatory indices and neurological outcomes of PAD was explored, and this can be validated in the future research.

## Conclusion

In summary, our study showed that elevated baseline NLR and NIHSS scores and aging may be independent risk factors for unfavorable prognosis in patients with PAD. NLR can be used as a potential biological indicator for predicting the prognosis of PAD, suggesting that intervening in the inflammatory response is expected to be a new target for preventing and treating stroke.

## Data availability statement

The raw data supporting the conclusions of this article will be made available by the authors, without undue reservation.

## Ethics statement

The studies involving humans were approved by the ethics committee of Suzhou Dushu Lake Hospital. The studies were conducted in accordance with the local legislation and institutional requirements. The participants provided their written informed consent to participate in this study.

## Author contributions

LS: Writing – original draft, Data curation, Formal analysis, Methodology, Software. XL: Data curation, Software, Writing – original draft. WH: Formal analysis, Software, Writing – original draft. LY: Data curation, Software, Writing – review & editing. SG: Data curation, Methodology, Writing – review & editing. YJ: Supervision, Visualization, Writing – review & editing. HP: Conceptualization, Supervision, Writing – review & editing. YH: Conceptualization, Visualization, Writing – review & editing.
