# Supplementary material for: Predictors for unfavorable prognosis after stroke with perforator artery disease
Source: Front Neurol. 2024 Jan 24;15:1340085. doi: 10.3389/fneur.2024.1340085 (PMC10847328; doi:10.3389/fneur.2024.1340085)
Supplement: Supplementary file 1 [file Table_1.DOCX]

Additional file 1: Table S1: ROC curves for unfavorable prognosis of patients with PAD

|  | AUC | *95%* CI | Optimal cutoff value | Specificity | Sensitivity | Youden index |
| --- | --- | --- | --- | --- | --- | --- |
| Age | 0.590 | 0.496-0.684 | 65.5 | 0.594 | 0.563 | 0.156 |
| NIHSS | 0.905 | 0.860-0.949 | 2.5 | 0.729 | 0.896 | 0.625 |
| NE | 0.636 | 0.538-0.734 | 4.990 | 0.782 | 0.500 | 0.282 |
| LYM | 0.768 | 0.693-0.843 | 2.005 | 0.632 | 0.833 | 0.344 |
| NLR | 0.798 | 0.727-0.869 | 2.712 | 0.692 | 0.771 | 0.463 |

（NIHSS: National Institute of Health stroke scale.NE: neutrophil. LYM: lymphocyte. NLR: neutrophil to lymphocyte ratio）
